# Local membrane deformation and micro-injury lead to qualitatively different responses in osteoblasts

**DOI:** 10.12688/f1000research.4448.1

**Published:** 2014-07-11

**Authors:** G. Monserratt Lopez-Ayon, Heng-Yen Liu, Shu Xing, Osama M. Maria, Jeffrey M. LeDue, Helene Bourque, Peter Grutter, Svetlana V. Komarova

**Affiliations:** 1 Center for the Physics of Materials and the Department of Physics, McGill University, 3600 University, Montreal, Quebec, H3A 2T8, Canada; 2 Faculty of Dentistry, McGill University, 3640 University, Montreal, Quebec, H3A 0C7, Canada; 3 Shriners Hospital for Children – Canada, 1529 Cedar Ave, Montreal, Quebec, H3G IA6, Canada

## Abstract

Micro-damage of bone tissue is known to regulate bone turnover. However, it is unknown if individual bone cells can differentiate between membrane deformation and micro-injury. We generated osteoblasts from mouse bone marrow or bone morphogenetic protein 2-transfected C2C12 cells. Single cells were mechanically stimulated by indentation with the atomic force microscopy probe with variable force load either resulting in membrane deformation only, or leading to membrane penetration and micro-injury. Changes in the cytosolic free calcium concentration ([Ca ^2+^] _i_) in fluo4-AM loaded cells were analyzed. When deformation only was induced, it resulted in an immediate elevation of [Ca ^2+^] _i_ which was localized to the probe periphery. Multiple consecutive local Ca ^2+^ responses were induced by sequential application of low level forces, with characteristic recovery time of ~2 s. The duration of [Ca ^2+^] _i_ elevations was directly proportional to the tip-cell contact time. In contrast, cell micro-injury resulted in transient global elevations of [Ca ^2+^] _i_, the magnitude of which was independent of the tip-cell contact time. Sequential micro-injury of the same cell did not induce Ca ^2+^ response within 30 s of the first stimulation. Both local and global Ca ^2+^elevations were blocked in Ca ^2+^-free media or in the presence of stretch-activated channel blocker Gd ^3+^. In addition, amount of Ca ^2+^ released during global responses was significantly reduced in the presence of PLC inhibitor Et-18-OCH _3_. Thus, we found qualitative differences in calcium responses to mechanical forces inducing only membrane deformation or deformation leading to micro-injury.

## Introduction

Mechanical stimulation of bone is well-known to regulate bone volume, structure and composition ^1, 2^. It was recently suggested that, in addition to deformation forces, microdamage plays an important role in regulating bone turnover and strength ^3^. Bone is restructured through the coordinated action of bone cells, osteoblasts responsible for bone formation and osteoclasts responsible for bone resorption. Cells of osteoblastic origin, including osteoblasts, osteocytes and bone lining cells are believed to act as mechanosensors in bone tissue ^1, 4^. Understanding how bone cells sense and react to mechanical forces is important for gaining insight into the mechanisms of bone adaptation to its mechanical environment.

Because of the complexity of the bone environment *in vivo*, several models have been developed to understand the effects of mechanical stimulation on bone cells *in vitro* ^5^. These models include application of hydrostatic pressure, longitudinal substrate stretch and fluid shear. These studies have established that osteoblastic cells can sense mechanical stimulation through plasma membrane receptors, stretch activated ion channels, as well as focal adhesion sites ^6^. Ca ^2+^ signaling was shown to be the prominent first response of osteoblastic cells to any type of mechanical stimulation ^7– 9^. Ca ^2+^ signaling induced by mechanical stimulation in turn influences numerous bone cell functions such as cytoskeletal reorganization ^7^, gene expression ^10^, proliferation and differentiation ^6^. However, these studies also identified significant complex signaling interactions between multiple cells ^6^, making it difficult to de-convolute the responses of single cells to mechanical stimulation. Moreover, these techniques do not allow exact control of forces applied to individual cells, nor do they report single cell micro-injury.

Local indentation techniques allow characterization of the response to mechanical stimulation at the single-cell level. Pipette microinjection ^11^, pipette suction ^11– 13^, and atomic force microscopy (AFM) ^14, 15^ have been used to study responses of individual osteoblasts to mechanical stimulation. From these techniques, only AFM allows application of precisely controlled forces in the nano-Newton scale, as well as provides readout of the extent of membrane deformation ^16^. Moreover, AFM can be used with cantilever tips of different geometries, which allow additional control of the distribution of the force. Spherical tip allows creating high range of membrane strains ^17^, while pyramidal tip allows reversible membrane penetration, which does not result in long-term cell damage ^18, 19^.

The goal of this study was to examine how a single osteoblastic cell responds to forces inducing cell membrane deformation only, or membrane deformation resulting in micro-injury. We used either primary bone marrow cells cultured with ascorbic acid, or C2C12 cells stably transfected with bone morphogenic protein (BMP) 2. C2C12 cells have been shown to undergo osteoblastic differentiation when treated with BMP-2 ^20^. Mechanical forces of different magnitude were applied using AFM. To monitor cell responsiveness to mechanical forces, changes in cytosolic free Ca ^2+^ concentration ([Ca ^2+^] _i_) were assessed.

## Materials and methods

### Cell cultures

All procedures were approved by McGill University’s Animal Care Committee (protocol number 2013-7332) and conformed to the ethical guidelines of the Canadian Council on Animal Care. Six week old male C57/BL6 mice (Charles River) were acclimatized for 1 week, fed ad libitum, and kept on a 12-hour light/dark cycle prior to euthanasia by CO _2_ asphyxiation followed by cervical dislocation. Femora and tibiae were isolated and separated from soft tissue. The bones were cut in two, placed in an Eppendorf tube, centrifuged twice at 10 ^3^ rpm for 30 seconds. Cells (~2×10 ^7^ cells/mouse) were re-suspended in Minimum Essential Medium (MEM (Eagle), from Invitrogen) supplemented with 2 mM of L-glutamine, 100 IU of penicillin, 100 μg/ml of streptomycin and 10% of fetal bovine serum (Wisent), and 5×10 ^6^ cells were plated on round 25 mm No.1 glass coverslips (Matteck Corporation), and cultured with 50 μg/ml of ascorbic acid at 5% CO _2_, 37°C for 4–6 days to 50–70% of confluence, which allowed easy identification of individual cells. The media was replaced every third day. The osteoblastic phenotype was confirmed by alkaline phosphatase staining (Fast Red, Sigma).

C2C12 cells stably transfected with BMP-2 (kindly provided by Dr M. Murshed, McGill University) were plated at 2.5×10 ^4^ cells on round 25 mm No.1 glass coverslips (Matteck corporation). Cells were cultured in Dulbecco's Modified Eagle Medium (DMEM, Invitrogen) supplemented with 2 mM of L-glutamine, 100 IU of penicillin and 100 μg/ml of streptomycin and 10% of fetal bovine serum at 5% CO _2_, 37°C to 50–70% of confluence. The media was changed every third day. The osteoblastic phenotype was confirmed by alkaline phosphatase staining.

### Intracellular calcium measurements

The cells were loaded with 1.5 μl of Ca ^2+^-sensitive dye fluo4–AM (Molecular Probes, stock solution of 1 mg/ml in DSMO), added to 2 ml of culture media for 40 minutes at room temperature. The cells were washed twice with physiological solution (130 mM NaCl; 5 mM KCl; 1 mM MgCl _2_; 1 mM CaCl _2_; 10 mM glucose; 20 mM HEPES, pH 7.4, for Ca ^2+^-containing experiments, or 0 mM CaCl _2_ and 10 mM EGTA for Ca ^2+^-free experiments) ^21^, the coverslip was assembled onto the peek fluid chamber (Asylum Research) and physiological buffered solution (1.5 ml) was added after loading with fluo4-AM. Gd ^3+^ (Sigma) was dissolved directly in buffer to 50 μM final concentration. 1-O-Octadecyl-2-O-methyl-sn-glycero-3-phosphorylcholine (Et-18-OCH _3_, Sigma) stock solution (5 mg/ml) was prepared in ethanol, which was used as a vehicle (0.07%) for corresponding experiments. Et-18-OCH _3_ was used at 5 μM final concentration. Cells were pretreated with inhibitors for 45 min at room temperature before mechanical stimulation. The cells were illuminated with 488 nm laser light and the emitted light was collected with the Cascade II camera. In each experiment a time-sequence of fluorescence images was acquired with one frame taken every 345 ± 10 ms with 250 ms exposure time. Traces were extracted from the video files using a code for Matlab 2011A provided in ^22^. The fluorescence signal in cells not exposed to mechanical stimulation did not demonstrate associated changes and was used as a reference for signal correction for bleaching and cantilever reflection artifacts using the protocol for data processing for systematic signal recovery that we established and described previously ^22^.

### Atomic force microscopy

The experiments were conducted using an MFP-3D-BIO AFM (Asylum Research, Santa Barbara CA) mounted on an Olympus IX-71 inverted optical microscope. The sample placed in the closed fluid cell was left undisturbed for 15 min to achieve thermal equilibrium at 37°C. A 60X oil immersion objective with 1.45 NA (Olympus) was put into contact with the coverslip allowing optical access from the bottom and AFM access on top of the sample. The region of interest was located and aligned with the cantilever tip using the bright field and fluorescence images.

### AFM probe preparation

The cantilever tip (NCLAuD, Nanosensors), was etched down to 1 μm ^2^ contact area with a focused ion beam microscope (FEI DB235). When sharp tips were used, the membrane rupture force event was not distinguishable in the force curve. Using Hooke’s law ( *F=k⋅d*), the force exerted by the probe ( *F*) was determined from the cantilever spring constant ( *k,* 39 ± 7.8 N/m) and deflection ( *d*). The net extension of the piezo element, the speed, the time of indentation, the time between indentations and the number of indentations were controlled using the Asylum MFP3D software in IgorPro 6.22 platform.

### Statistical analysis

Data are presented as representative images, representative traces, means ± SE with n being the number of experiments analyzed. The normalized amplitude as a function of time, the amplitude decay rate as a function of recovery time and the response duration as a function of the contact time were analyzed using least squares regression and fitted a line or an exponential as appropriate. Categorical data were analyzed as described previously ^23^. Statistical differences were assessed using Fisher Exact Probability test for categorical data or Student’s t-test for continuous data and were accepted as significant at p < 0.05. Statistical analysis was performed in Microsoft Excel 2007.

## Results

Data on the effects of local membrane deformation and micro-injury in osteoblasts The dataset consists of the following: Force-distance curves collected during AFM application using Asylum MFP3D software in IgorPro 6.22 platform, labeled as “Force curves” Video files of changes in fluorescence intensity of fluo-4 loaded cells during the indentation experiment labeled as “movie” Examples of traces of changes in fluorescence intensity extracted from the video files using a code for Matlab 2011 A available in reference 22 in the associated article, labeled as “Mean Ca Fluorescence intensity”

### Experimental setup

**Figure 1.**
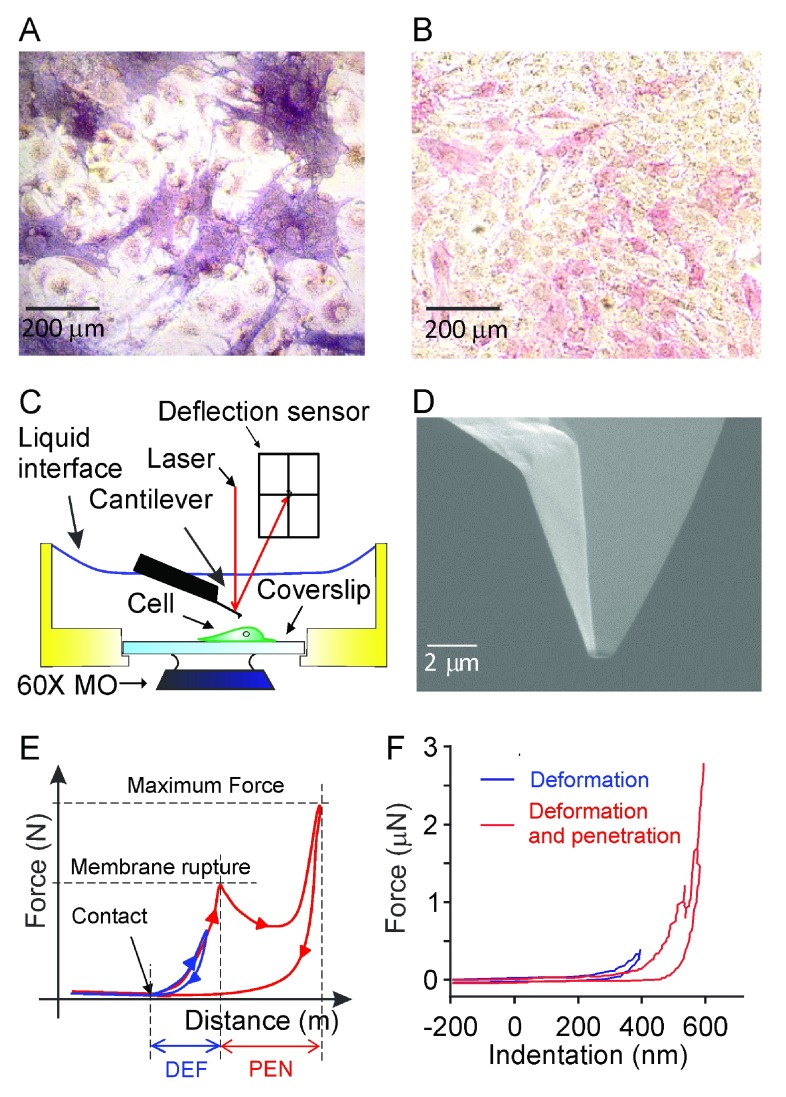
Mechanosensitivity of osteoblastic cells assessed by atomic force microscopy (AFM). Bone marrow cells were cultured in the presence of ascorbic acid (50 μg/ml) for 4–6 days to induce osteoblast differentiation or C2C12 cells stably transfected with BMP-2 were cultured for 2–6 days to obtain osteoblastic phenotype. **A**, **B**) Representative images of bone marrow culture on day 5 ( **A**) and C2C12 culture on day 3 ( **B**) stained for osteoblastic marker, alkaline phosphatase (red). **C**) Schematic representation of the AFM operation under liquid: the sample and the tip are submerged and changes in the laser reflection signal due to deflection of the cantilever are monitored. **D**) Experiments were performed using a probe with 1 μm ^2^ tip area; the tip is etched using focused ion beam. **E**) Schematic representation of events observed using force-distance curve: *a)* as the AFM tip approaches the cell, the *contact* point is evident as increase in force required to move the probe; *b)* when the probe indents the cell membrane, the force increases until it reaches the *membrane rupture* force and the tip penetrates the cell (apparent by a decrease in the cell resistance); *c)* indentation is continued until a predetermined *maximum force* is reached, and the probe retraction is initiated. The retraction curve commonly deviates from the approach curve, reflecting that energy is required for deformation and penetration of the cell membrane. The maximum force was set to be either below (blue) or above (red) the membrane rupture force. **F**) The example force-distance curves from the experiments in which deformation only (blue) or deformation plus penetration (red) were induced.

We employed two cell models to study osteoblast mechanosensitivity: *i*) mouse bone marrow cells cultured in the presence of ascorbic acid (50 mg/ml) for 4–6 days ( Figure 1A) or *ii*) C2C12 cells stably transfected with BMP-2 and cultured for 2–6 days ( Figure 1B). Both models represent osteoblastic cells at the early differentiation stage due to the limitation of AFM use in confluent and multilayered cultures. Osteoblastic phenotype was confirmed in fixed cultured by alkaline phosphatase staining. In parallel live cultures, the cells exhibiting osteoblastic morphology (strongly adhered cells with relatively large body and several filopodia) were chosen for mechanical testing. Mechanical stimulation was performed with AFM force spectroscopy indentation ( Figure 1C) using a 1 μm ^2^ area tip ( Figure 1D). Using AFM allows strict control of the amount of force applied to the cell ( Figure 1E, F). The membrane penetration event is easily identifiable on the force-distance curve as a decrease in the force required to continue to move the probe. The membrane penetration force was found to be similar for osteoblasts obtained in different cultures: 516 ± 200 nN for primary osteoblasts and 672 ± 100 nN for C2C12 osteoblasts. We analyzed two distinct modes of mechanical stimulation: *i)* the maximum force was set below the membrane penetration force (at ∼400 nN), leading only to membrane deformation (low-load, Figure 1E, F, *blue*); and *ii)* the maximum force was set above the membrane penetration force (∼2800 nN), inducing membrane rupture and penetration (high-load, Figure 1E, F, *red*).

### Changes in [Ca ^2+^] _i_ induced by different indentation regimes

**Figure 2.**
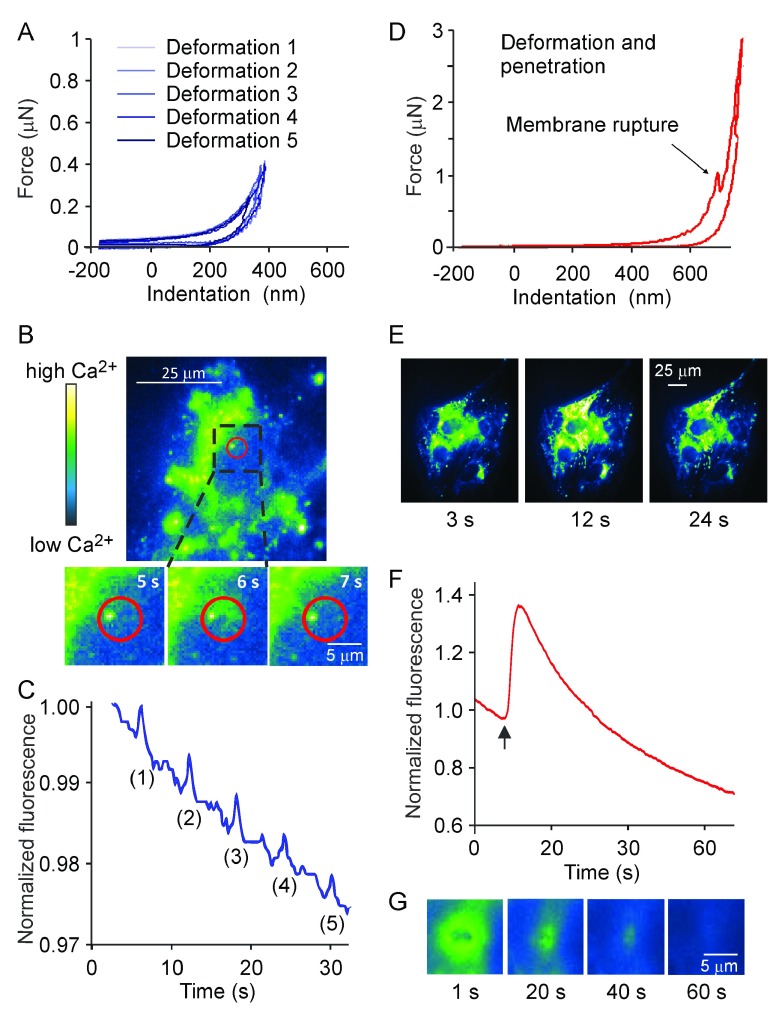
Low level membrane deformation induces local Ca ^2+^ response, whereas deformation leading to membrane micro-injury induces global elevations of [Ca ^2+^] _i_. Osteoblasts generated from C2C12 cells were loaded with Ca ^2+^-sensitive dye fluo4-AM and changes in [Ca ^2+^] _i_ in response to mechanical stimulation were assessed. **A–C**) The maximum force was set below the membrane rupture force. **A**) Representative force-distance curves depicting multiple local indentations of the cell membrane. **B**) A single cell exhibiting local [Ca ^2+^] _i_ elevation in response to membrane deformation. Magnified below is the region of the cell centered at the indentation point, that demonstrates the changes in [Ca ^2+^] _i_ with time following a single AFM indentation at 5 s. In pseudo-color, black/blue represents low and yellow/white represents high [Ca ^2+^] _i_ levels. **C**) Representative trace depicting changes in fluorescence intensity in the region shown in red on **B**, in response to multiple membrane deformations. **D–G**) The maximum force was set above the membrane rupture force. **D**) Force-distance curve demonstrating the membrane deformation and penetration. **E**) Micrographs demonstrating global increase in fluorescence intensity following a single high-load indentation at 6 s (same color scale as in **B**). **F**) Changes in fluorescence intensity in the whole cell shown in **E** following single indentation with the penetration of cell membrane (indicated by an arrow). **G**) Magnified region of the cell centered at the high-load indentation point, demonstrating the transient character of membrane damage.

Since [Ca ^2+^] _i_ elevations are known to be the most common first responses to mechanical stimulation ^4, 9, 11, 24^, we analyzed the changes in [Ca ^2+^] _i_ in response to different loading regimes in the osteoblasts loaded with [Ca ^2+^] _i_ fluorescent indicator fluo4-AM. In the low load indentation regime, where the force exerted on the cell induced membrane deformation ( Figure 2A), only local [Ca ^2+^] _i_ transients were observed ( Figure 2B). To analyze the transients, the spatially-averaged intensity over a circle of 5 μm in diameter centered on the point of indentation ( *red circle* in Figure 2B) was normalized to the initial baseline signal and plotted as a function of time ( Figure 2C). When larger circles were taken, the change in averaged intensity was smaller; if the whole cell was selected, the local response was not apparent. When consecutive low load indentations were performed at the same location on the same cell multiple local responses were induced ( Figure 2A, C).

In the high load regime, which resulted in local membrane rupture and penetration of the cell ( Figure 2D), whole cell [Ca ^2+^] _i_ elevations were observed ( Figure 2E). When the normalized fluorescence intensity was averaged over an ellipse surrounding the whole cell and plotted as a function of time ( Figure 2F), it was apparent that global [Ca ^2+^] _i_ elevations were transient. When we focused on the membrane level to directly visualize the closure of the membrane micro-injury, we observed that it seals within 40–60 s after the stimulation ( Figure 2G). It is conceivable that high load indentation may affect the cell viability. However, in all our experiments, the cell previously exposed to high load indentation maintained basal calcium levels within normal range for the whole period of observation (5–30 min), and when directly tested using Trypan Blue exclusion test, six out of six cells previously indented were viable ∼10 minutes after high load indentation. Taken together, these data strongly suggest that the micro-injury induced by AFM probe is reversible and does not result in cell death.

Since application of AFM may potentially interfere with fluorescence measurements, we have previously performed in depth analysis of the system performance ^22^. We have found that in experiments where local repetitive stimulations were performed, reflection artifacts correlated with cantilever motion represented a significant component of the fluorescent signal. We developed a protocol to correct the fluorescence traces for reflection artifacts, as well as photobleaching ^22^. The traces were processed as follows: 1) the region of interest was selected in the digital image; 2) the average fluorescence intensity data were extracted and normalized to the initial basal reading to correct for differences in dye loading; 3) the signal was corrected for bleaching and reflection artifacts as described in ^22^; 4) the noise was determined as the standard deviation in a linear portion of the trace, and 5) the [Ca ^2+^] _i_ response was deemed positive when an increase in intensity exceeded four-fold the average noise. Cells exhibiting spontaneous [Ca ^2+^] _i_ fluctuations ^25^ were excluded from the study.

### Characterization of local [Ca ^2+^] _i_ responses induced by membrane deformation

**Figure 3.**
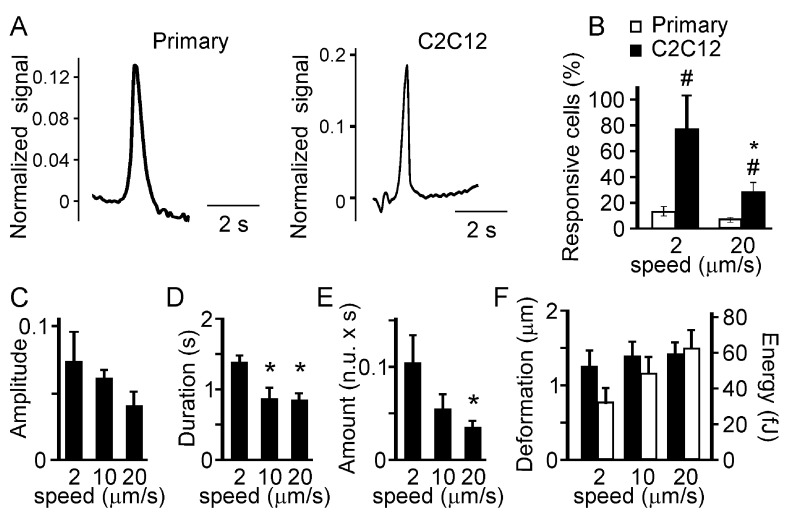
Characteristics of the local Ca ^2+^ responses. Primary or C2C12 derived osteoblasts were loaded with fluo4-AM and single indentations were performed at different speeds (2, 10, 20 mm/s). The maximum force was set below the membrane rupture force. **A**) The examples of local Ca ^2+^ elevations induced by a low level deformation in a primary osteoblast ( *left*) and a C2C12-derived osteoblast ( *right*). **B**) The percentage of primary (white) or C2C12-derived (black) osteoblasts responding to deformations with local Ca ^2+^ elevations. Data are means ± SE, n = 15 for primary osteoblasts, n=10 and 30 for C2C12 osteoblasts deformed at 2 and 20 μm/s respectively, # indicates p<0.05 difference between primary and C2C12-derived osteoblasts, * indicates p<0.05 difference between deformation speeds, assessed by Fisher Exact Probability test for categorical data. **C–E**) Local Ca ^2+^ transients in C2C12-derived osteoblasts were analyzed for the amplitude of Ca ^2+^ response ( **C**), the duration of Ca ^2+^ response ( **D**) and the amount of Ca ^2+^ released during the response ( **E**). Data are means ± SE; n is the number of responses from 10 trials: for 2 µm/s n = 6, for 10 µm/s n = 2, for 20 µm/s n = 6, *indicates p<0.05 difference as assessed by Student’s t-test. **F**) Force-distance curves were analyzed and the extent of membrane deformation (black, scale on the left) and energy spent to deform the membrane (white, scale on the right) were assessed. Data are means ± SE, n = 5, no significant difference.

First, we compared the responses in C2C12-derived and primary osteoblasts. We have found that the low load indentation induced qualitatively similar responses in the primary and C2C12-derived osteoblasts ( Figure 3A). However, when we compared the percentage of cells exhibiting local [Ca ^2+^] _i_ transients in response to indentation performed at low and high speeds, we found that a higher percentage of C2C12-derived osteoblasts responded to the mechanical stimulation compared to primary osteoblasts ( Figure 3B). In addition, a higher percentage of C2C12-derived osteoblasts exhibited [Ca ^2+^] _i_ transients when indented at lower speed compared to higher speed ( Figure 3B). In primary osteoblasts a similar trend was observed, however it did not reach statistical significance since the rate of response was low in these cells. We analyzed in depth the [Ca ^2+^] _i_ responses induced in C2C12-derived osteoblasts by a single low-load indentation performed at different speed (2, 10 and 20 μm/s). The [Ca ^2+^] _i_ elevations were further characterized by measuring *i*) the relative amplitude of the response (with respect to basal); *ii*) the duration of the response (the width of a transient at the half maximum amplitude); and *iii*) the amount of [Ca ^2+^] _i_ (duration multiplied by amplitude). The speed of indentation did not significantly affect the amplitude of calcium responses ( Figure 3C). However, indentations performed at low speed induced calcium responses of longer duration ( Figure 3D) and thus resulted in release of greater amount of [Ca ^2+^] _i_ ( Figure 3E) compared to indentations performed at high speed. To analyze if indenting at different speeds delivered different mechanical stimulation, the force curves were characterized by quantifying *i*) the extent of membrane deformation (the distance between the contact point and the maximum piezo extension) ( Figure 3F), and *ii*) the energy spent to deform the cell (the area between the approach and retraction curves) ( Figure 3F, right scale). No statistically significant difference was observed in these parameters when different indentation speeds were compared.

**Figure 4.**
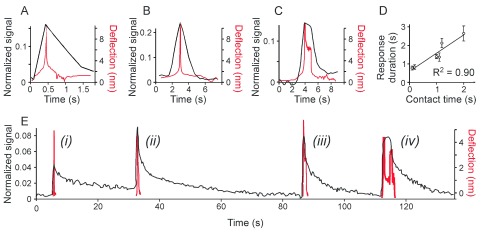
The duration of the low level deformation determines the duration of the Ca ^2+^ elevation. C2C12-derived osteoblasts were loaded with fluo4-AM and responses to single indentation were assessed. The maximum force was set below the membrane rupture force. **A–C**) Examples of Ca ^2+^ transients in black (left scale) in response to indentation performed with different contact time. Overlaid are the cantilever deflection curves in red (right scale). The indentation speed was set to 20 mm/s ( **A**) or 2 mm/s ( **B, C**). For **C**, the probe was maintained in contact with the cell for additional 1 s. **D**) The Ca ^2+^ transient duration is plotted as a function of contact time. Data are means ± SE, for 0.1 s and 1.2 s n = 4, for 0.2 s n = 2, for 1.0, 1.1 and 2.0 s n = 3. Linear fit is *f(x) = 0.98x + 0.60* with R ^2^=0.90. **E**) A single cell exhibited [Ca ^2+^] _i_ elevations of different duration in response to indentations with different contact times. The indentation speed was 20 µm/s, the probe was maintained in contact with the cell for additional *i*) 0 s, *ii*) 0.3 s, *iii*) 0.7 s and *iv*) 3 s.

We next assessed how the contact time (the net time the probe spent in contact with the cell during stimulation) affects calcium responses to low-load indentations performed at different speeds. We varied the contact time by increasing by 1 s the time that the tip spent in the maximally extended piezo position. The reflection of a cantilever on the calcium fluorescence recording allows achieving exact temporal overlay between the fluorescence intensity and the force-distance curve (as described in ^22^). The [Ca ^2+^] _i_ signal was initiated when the tip came into contact with the cell and was maximum at the maximum deflection ( Figure 4A, B). When the contact time was increased by 1 s, the comparable increase in calcium response duration was observed ( Figure 4C). The mean of the response duration was plotted as a function of the corresponding contact time demonstrating that the average calcium response duration was directly proportional to the contact time, R ^2^ = 0.90 ( Figure 4D). We next assessed if calcium responses of different durations can be induced in a single cell by varying the contact time (as the piezo extension time) in consecutive indentations ( Figure 4E). We have found that a single cell exhibited [Ca ^2+^] _i_ elevations of different durations ( Figure 4E, *i-iv, black*) when indentations with different contact times ( Figure 4E, *i-iv, red*) were performed.

**Figure 5.**
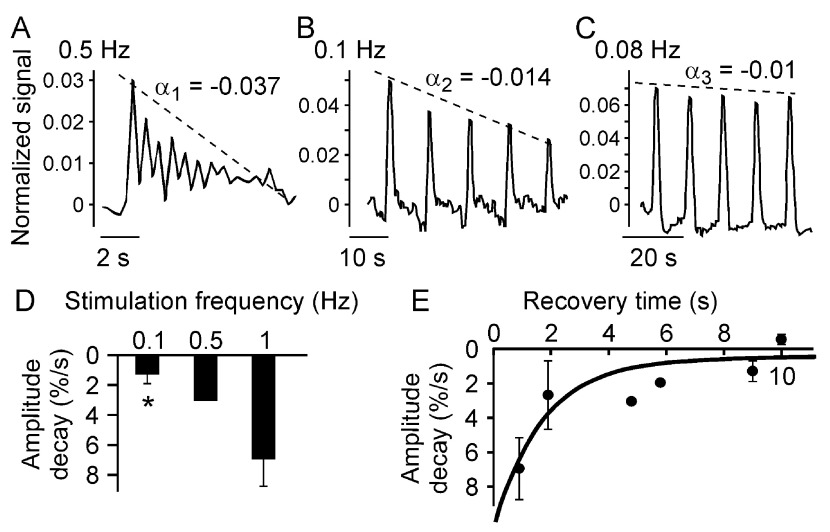
Multiple consecutive low level indentations induce multiple cell responses. C2C12-derived osteoblasts were loaded with fluo4-AM and changes in [Ca ^2+^] _i_ in response to multiple indentations were assessed. The maximum force was set below the membrane rupture force. **A–C**) Examples of multiple Ca ^2+^ transients in response to consecutive indentations performed at 10 µm/s, 0.5 Hz ( **A**), 2 µm/s, 0.1 Hz ( **B**) and 2 µm/s, with a 2 s delay between stimulations, 0.08 Hz ( **C**). The amplitudes of the consecutive responses were normalized with respect to the first response and the amplitude decay rate α _*i*_ was determined as the slope of a linear fit to the amplitude-time graph (dashed line). **D**) The amplitude decay rates were plotted as a function of the frequency of indentation. Data are means ± SE (except for 0.5 Hz), n is a number of responses from 5 trials: for 0.1 Hz n = 4, for 0.5 Hz n = 1, for 1 Hz n = 4, *indicates p<0.05 difference compared to 1 Hz, assessed by Student’s t-test. **E**) The amplitude decay rates were plotted as a function of the recovery time. Data are means ± SE (except for 4.8 and 5.8 s), n is a number of responses from 5 trials: for 0.9 s n = 3, for 1.9 s n = 4, for 4.8 s and 5.8 s n = 1, for 9 s n = 4 and for 10 s n = 3. The solid line represents an exponential fit: *f(x) = a exp(-x b)+c* with a time constant of b = 0.5s ^-1^; τ~2s, RMSE = 0.02 s ^-1^ (R ^2^=0.77).

Since we have found that repeated low level mechanical stimulations can elicit multiple consecutive responses in a single cell, we next examined how the exposure to mechanical stimulation affects subsequent responses of the same cell. Multiple low load indentations were performed on the same cell with different frequencies, and [Ca ^2+^] _i_ changes in response were assessed ( Figure 5). We have found that when cells were stimulated with higher frequency, the amplitude of the response in consecutive stimulations decreased ( Figure 5A–C). Within each sequence of Ca ^2+^ responses to the consecutive indentations, we normalized the amplitudes with respect to the amplitude of Ca ^2+^ response to the first indentation and quantified the amplitude decay rate as a slope (α _*i*_) of the best fit line for the responses. This slope is negative, representing a decrease in the amplitude, and when multiplied by 100, this slope is expressed as percentage decay. We have found that amplitude decay is significantly higher when cells are stimulated with higher frequencies ( Figure 5D). At different frequencies two parameters are changing – the contact time, defined as the time the probe spends in contact with the cell, and recovery time, defined as the time off contact between stimulations. We next specifically varied the recovery time by introducing a delay of 1 s between consecutive stimulations, and plotted the average amplitude decay rates as a function of the recovery time ( Figure 5E). An exponential curve with a characteristic recovery time of 2.00 ± 0.08 s was found to be the best fit for the relationship between the amplitude decay rate and the recovery time. These data suggest that a refractory period of ~10 s is required for complete amplitude recovery after low load mechanical stimulation.

### Characterization of global [Ca ^2+^] _i_ responses induced by membrane deformation and micro-injury

**Figure 6.**
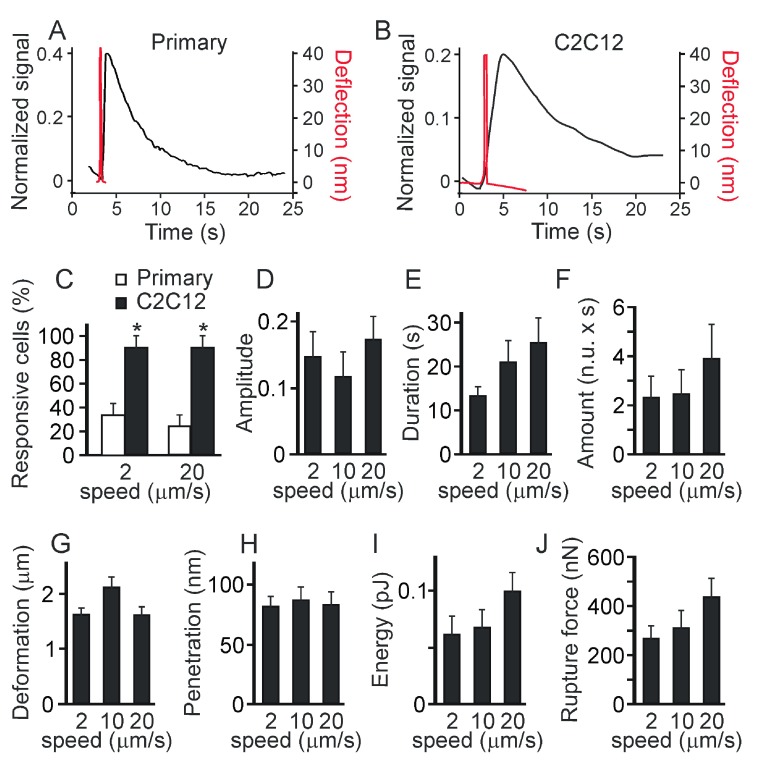
The Ca ^2+^ responses to indentation resulting in penetration are not significantly affected by the speed of the indentation or the contact time. Primary mouse osteoblasts or osteoblasts generated from C2C12 cells were loaded with fluo4-AM and single indentations were performed. The maximum force was set above the membrane rupture force. **A, B**) Examples of Ca ^2+^ transients (left scale) in primary osteoblast ( **A**) or C2C12-derived osteoclast ( **B**) in response to the high-load indentations. Overlaid are the cantilever deflection curves in red ( *right scale*). **C**) The percentage of cells that exhibit global Ca ^2+^ elevations in primary (white) or C2C12-generated (black) osteoblasts in response to the high-load indentations performed with different speed of 2 or 20 μm/s. Data are means ± SE, n = 25 for primary osteoblasts and n=10 for C2C12-derived osteoblasts, # indicates a p<0.05 difference between primary and C2C12-generated osteoblasts as assessed by Fisher Exact Probability test. **D–F**) Changes in global Ca ^2+^ in response to membrane penetration in C2C12-derived osteoblasts were analyzed for the amplitude of Ca ^2+^ response ( **D**), the duration of Ca ^2+^ response ( **E**) and the amount of Ca ^2+^ released during the response ( **F**). Data are means ± SE, n is the number of response from 10 trials: for 2 µm/s n = 9, for 10 µm/s n = 10, and for 20 µm/s n = 9, no significant difference. **G**– **J**) Force-distance curves were analyzed for the extent of membrane deformation ( **G**), penetration depth ( **H**), energy spent to deform and penetrate the membrane ( **I**) and membrane rupture force ( **J**). Data are means ± SE, n = 10, no significant difference.

When the maximum force was set at 2800 nN (three-fold above the membrane penetration force), the force-distance curves confirmed that membrane penetration occurred in all the cells tested. We have found that qualitatively similar responses were induced in primary and C2C12-derived osteoblasts ( Figure 6A, B), however the response rate was significantly lower for the primary osteoblasts ( Figure 6C). When the whole cell average fluorescence intensity and the force-distance curves of individual cells were overlaid in time, we found that, in contrast to low-load indentation, micro-injury-induced global [Ca ^2+^] _i_ elevations were delayed with respect to the maximum deflection of the probe (which indicates maximum cell penetration), and reached their amplitude after the tip was released from cell contact ( Figure 6A, B). We assessed [Ca ^2+^] _i_ changes induced in C2C12-derived osteoblasts by a single high-load indentation performed at different speeds (2, 10 and 20 μm/s). There was no significant correlation between the indentation speed and the amplitude ( Figure 6D), duration ( Figure 6E) or amount of Ca ^2+^ released ( Figure 6F) during the responses. The force-distance curve analysis did not demonstrate statistically significant differences in the extent of membrane deformation prior to the membrane penetration ( Figure 6G), extent of membrane penetration ( Figure 6H), energy spent in deformation, micro-injury and penetration ( Figure 6I) or membrane rupture force ( Figure 6J).

### Contribution of mechanosensitive channels and calcium stores to elevations in [Ca ^2+^] _i_ induced by membrane deformation and micro-injury

**Figure 7.**
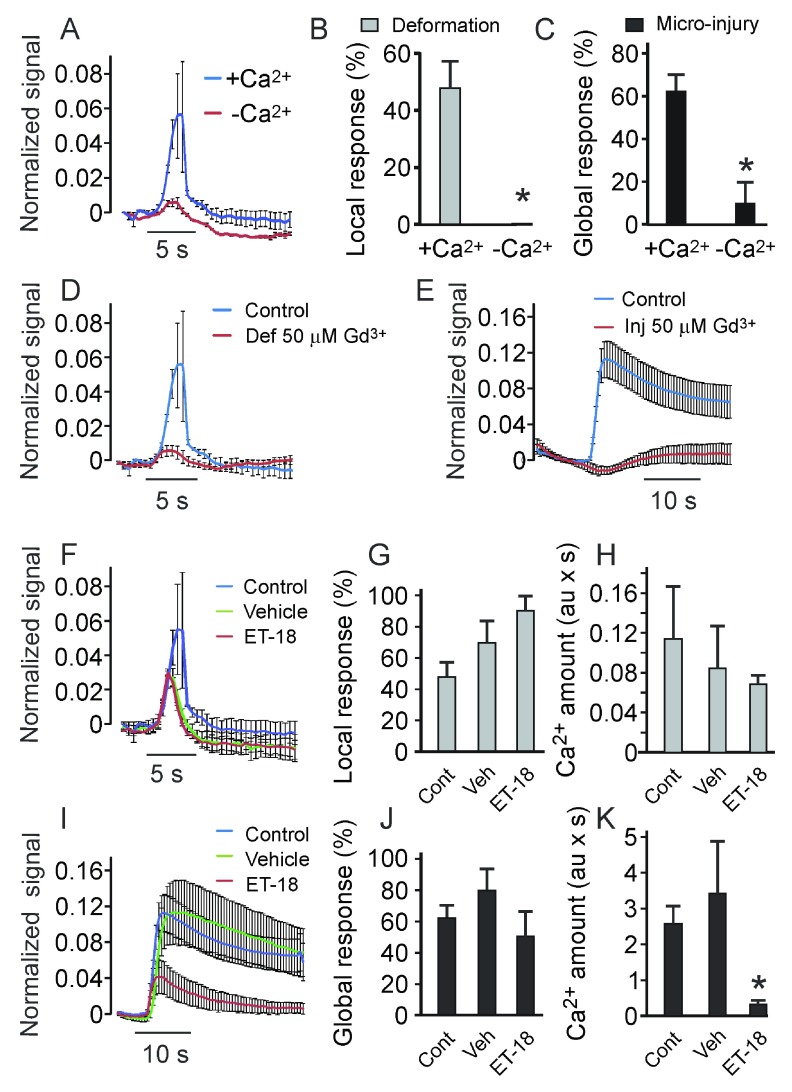
The membrane deformation-induced Ca ^2+^ responses are affected by inhibitors of stretch-activated Ca ^2+^ channels and phospholipase C. C2C12-generated osteoblasts were loaded with fluo4-AM, and single indentations were performed at 2 μm/s. **A**) Average local Ca ^2+^ transients in response to low-load indentation in Ca ^2+^-containing (blue) and Ca ^2+^-free (10 mM EGTA, red) buffer. Data are means ± SE. **B**, **C**) Percentage of cells exhibiting local ( **B**) and global ( **C**) Ca ^2+^ elevations in Ca ^2+^-containing (+Ca ^2+^) or Ca ^2+^ free (-Ca ^2+^) buffer in response to low-load ( **B**) or high-load ( **C**) indentation. Data are means ± SE, for ( **B**) n = 30 for Ca ^2+^-containing, n = 10 for Ca ^2+^-free; for ( **C**) n = 10; *indicates a p<0.05 difference as assessed by Fisher Exact Probability test. **D**) Average local Ca ^2+^ transients in response to membrane indentation in control (blue) or mechanosensitive Ca ^2+^ channel blocker Gd ^3+^ (50 µM)-containing buffer (red). Data are means ± SE. **E**) Average global Ca ^2+^ transients in response to membrane penetration in control (blue) or Gd ^3+^-containing buffer (red). Data are means ± SE. **F**) Average local Ca ^2+^ responses to membrane indentation in control (blue), vehicle (0.07% ethanol)-containing (green) or PLC inhibitor Et-18-OCH _3_-containing (red) buffer. Data are means ± SE. **G**) The percentage of cells that exhibited local Ca ^2+^ elevations in response to membrane indentation in control, vehicle- or Et-18-OCH _3_-containing buffer. Data are means ± SE, n = 30 for control, n = 10 for vehicle and Et-18-OCH _3_-treated cells, no significant difference. **H**) Average amount of Ca ^2+^ released in response to membrane indentation in control, vehicle- or Et-18-OCH _3_-containing buffer. Data are means ± SE, n = 4–5, no significant difference. **I**) Average global Ca ^2+^ elevations in response to membrane penetration in control (blue), vehicle-containing (green) or Et-18-OCH _3_-containing (red) buffer. Data are means ± SE. **J**) The percentage of cells that exhibited global Ca ^2+^ elevations in response to membrane penetration. Data are means ± SE, n = 33 for control, n = 10 for vehicle and Et-18-OCH _3_-treated cells, no significant difference. **K**) Average amount of Ca ^2+^ released in response to membrane penetration. Data are means ± SE, n = 8 for control and vehicle, n = 5 for Et-18-OCH _3_-treated cells, *indicates a p<0.05 difference as assessed by Student’s t-test.

Since the rise in [Ca ^2+^] _i_ can be due to influx from the extracellular space as well as to release from intracellular stores, we next investigated the contribution of these pathways to membrane deformation and to micro-injury-induced [Ca ^2+^] _i_ responses. We analyzed changes in [Ca ^2+^] _i_ in response to low-load and high-load mechanical stimulations in C2C12 osteoblasts maintained in the control Ca ^2+^-containing buffer or in Ca ^2+^-free, EGTA (10 mM)-containing buffer ( Figure 7A–C). Both local [Ca ^2+^] _i_ elevations ( Figure 7A, B) and global [Ca ^2+^] _i_ responses ( Figure 7C) were prevented by the lack of calcium in the extracellular space. We next inhibited mechanosensitive membrane channels using Gd ^3+^ (50 μM) ^17^. In the presence of Gd ^3+^ both local [Ca ^2+^] _i_ transients in response to membrane deformation ( Figure 7D) and micro-injury-induced global [Ca ^2+^] _i_ responses ( Figure 7E) were prevented. To inhibit the phospholipase C (PLC)-inositol triphosphate (IP _3_) pathway leading to calcium release from intracellular stores, we used PLC inhibitor Et-18-OCH _3_ (5 μM) ^17^. In the low-load regime, local [Ca ^2+^] _i_ transients ( Figure 7F) occurred at the same frequency as in control ( Figure 7G), and the amount of Ca ^2+^ released during the responses was not significantly affected ( Figure 7H). However, global [Ca ^2+^] _i_ transients induced by the high-load stimulation were noticeably smaller in Et-18-OCH _3_-treated, but not in vehicle-treated cells compared to control ( Figure 7I). The percentage of cells exhibiting global [Ca ^2+^] _i_ transients in response to membrane penetration was not significantly affected by Et-18-OCH _3_ or vehicle ( Figure 7J). However, the amount of Ca ^2+^ released during the response was significantly decreased in cells treated with Et-18-OCH _3_ ( Figure 7K). These data indicate that mechanosensitive Ca ^2+^ channels mediate both local and global responses, while the intracellular Ca ^2+^ stores are important for global responses only.

## Discussion

In this study, we have examined the ability of individual osteoblasts to respond to mechanical stimulation applied using AFM. Variable force load was applied resulting in either membrane deformation only or in membrane penetration and micro-injury, and changes in [Ca ^2+^] _i_ in fluo-4 loaded cells were analyzed. Qualitatively different Ca ^2+^ responses were observed in different force loads. In response to membrane deformation only, immediate local [Ca ^2+^] _i_ elevations limited to the indentation region were observed. Multiple stimulations of a single cell resulted in Ca ^2+^ responses of similar amplitude if a recovery time of more than 2 s between the stimulations was allowed. The duration of Ca ^2+^ responses to low-load indentation was proportional to the duration of deformation. In contrast, micro-injury induced global Ca ^2+^ elevation, which continued to develop after the removal of the probe and was independent of the duration of indentation. These data demonstrate that Ca ^2+^ responses to local membrane deformation exhibit threshold properties when micro-injury is induced.

In our model, micro-injury was required in order to achieve global elevations of [Ca ^2+^] _i_ in the majority of cells. Many techniques used to study cell mechanosensitivity, such as fluid flow, substrate strain and pipette indentation, do not allow sufficient resolution to detect membrane micro-injury. However, a number of studies, in which the absence of membrane penetration can be reliably confirmed, demonstrated that global Ca ^2+^ responses can be induced by membrane deformation only ^11, 17^. Several differences in the experimental setup could account for this discrepancy. Charras and Horton ^17^ used AFM with spherical tip of 10–30 μm diameter, which would deform a 100-fold larger area compared to the pyramidal tip with 1 μm ^2^ contact area used in our study. Thus, the extent of horizontal membrane involvement in the deformation may be important for cell mechanosensitivity. While Xia and Ferrier ^11^ used patch-clamp micropipette with similar dimensions to the pyramidal tip used in our study, it has been shown that pipette suction creates substantially larger strains compared to AFM micro-indentation ^14^. Therefore, higher calcium responses observed in the Xia and Ferrier study compared to our study are in keeping with previously demonstrated critical role of vertical membrane deformation in cell mechanosensitivity ^17^. We have now shown that the duration of Ca ^2+^ responses is directly proportional to time the cell membrane spends in deformation. Therefore, it is likely that cell sensitivity to membrane deformation is related to a combined effect of: *i*) the extent of the horizontal involvement of the membrane, *ii*) the vertical deformation of the membrane and *iii*) the duration of the deformation. Mechanosensitive calcium channels were strongly implicated in generating Ca ^2+^ responses to mechanical stimulations in previous studies ^11, 17, 25^ and were confirmed to provide a critical contribution to both local and global Ca ^2+^ responses in our study. Therefore, it is possible that local intracellular Ca ^2+^ acts as an integrating signal that increases when open channels are more numerous or are activated longer, until it reaches the threshold Ca ^2+^ concentration necessary to induce global Ca ^2+^ response. Micro-injury then contributes to increasing local Ca ^2+^ levels to threshold concentrations, inducing global response. Once global calcium elevation is triggered, it proceeds independently of the level of mechanical stimulation, as suggested by the lack of correlation between the magnitude of global Ca ^2+^ response and mechanical stimulation in our study and that of Charras and Horton ^17^. Our data demonstrate that calcium release from intracellular stores plays an important role in this process.

We identified the refractory periods during which the responses to subsequent mechanical stimulations were either absent or diminished in amplitude. This refractory period following membrane deformation only was relatively short, and the calcium response was fully reestablished after the recovery time of 10 s. In contrast, in experiments where micro-injury and global elevation of calcium were observed, the recovery period was longer than 30 s. Previously, the refractory periods for global Ca ^2+^ responses to fluid flow were reported to be in the order of 600–900 s ^26^. The presence of refractory periods is of potential importance, since it was shown previously, that in order to induce potent osteogenic response, mechanical loading of bone should be performed as a series of repeated loading periods separated by the periods of rest ^27, 28^. Of interest, the rest period of 10 s was found to be sufficient to induce potent bone formation in response to low magnitude mechanical loading ^28, 29^.

Taken together, our study provides new insights into the complex dynamics of cellular responses to mechanical stimulations. In contrast to many previous studies of cell responses to mechanical forces, atomic force microscopy allows very precise control and monitoring of the physical parameters of the experiment, such as forces and deformations applied at a single cell level. Using a well-established readout of cellular response, calcium signaling, allowed us to identify novel correlates between mechanical stimulation and cell responses. Such knowledge is important for better understanding of the mechanisms of mechanical loading-induced bone formation, as well as micro-damage induced bone remodeling.

## Data availability

figshare: Data on the effects of local membrane deformation and micro-injury in osteoblasts, doi: http://dx.doi.org/10.6084/m9.figshare.1091356 ^30^
