# Supplementary material for: Local membrane deformation and micro-injury lead to qualitatively different responses in osteoblasts
Source: F1000Res. 2014 Jul 11;3:162. [Version 1] doi: 10.12688/f1000research.4448.1 (PMC4168753; doi:10.12688/f1000research.4448.1)
Supplement: Peer review history

# Peer review: Referee response for version 1

**DOI:** 10.5256/f1000research.4759.r5751

**Published:** 2014-09-03

**Authors:** Daniel Isabey, Minh Nguyen

**Affiliations:** 1 Cell and Respitory Biomechanics, French Institute of Health and Medical Research, Paris, France; 2 Inserm, Paris, France

The article by Lopez-Ayon *et al.* compares the calcium (Ca ^2+^) response of osteoblasts to mechanical forces resulting from 2 types of well-defined micro-damages, namely a local membrane deformation and a micro-injury. An atomic force microscope is advantageously used to control and modulate these 2 types of stretching effects. In its present form, the article is already well structured, rich in new experimental results and easy to read. However, their message would be more complete if authors were considering the following points.

**Major comments**

Concerning methodological aspects:

1. Since the probe calibration is very important for AFM measurements, authors should provide more details concerning this indispensable step. For instance, the method used to determine the spring constant of the probe is never mentioned.
2. There is a non negligible hydro-drag force applied to the cantilever when it is moved at speed of 20 µm/s. If this aspect is not taken into account, the effective forces applied to cells and the energy spent to deform the membrane may be overestimated. A comment should be provided concerning this point.
3. The method used to quantify the Ca ^2+^ concentration is based on the analysis of averaged intensity in a predetermined zone drawn on the images acquired by fluorescent microscopy. So, signal intensity could depend on the layer which is in focus. The increase in intensity might thus be to the fact that cell is locally compressed leading to the generation of a calcium flow toward the basal cell face which is in focus. This additional effect should be appropriately discussed.

 

 Concerning Results and Discussion:

1. The authors write in page 4 that: "When consecutive low load indentation were performed at the same location on the same cell multiple local responses were induced". This results is indeed comforted by the calcium concentration images shown at 6 s in Fig 2B. Multiple calcium spots are visible at 6 s on the non magnified cellular image, suggesting that a local mechanical effect may induce multiple (and durable) distant yet local effects. Such a mechanically induced signaling at a distance has been largely described and commented by the group of Ning Wang presently working at University of Illinois at Urbana Champaign (e.g., Wang *et al.,* 2009). Clearly a tensed cytoskeleton plays a key role in such a distant phenomena and using, e.g., cytochalasin D, would have been of interest to prove that the distant local effects are related to the tensed cytoskeleton. Authors should at least comment on this interesting result revealed by the local membrane deformation test. Such an distant effect is not so evident in case of membrane rupture suggesting (since mechanical effect become global) important modifications for the cytoskeleton between the two types of stress studied. These distant effects could advantageously be analyzed and quantified.
2. It is not clear whether the cells are tested in isolated conditions (like in Fig 2B) or with some degree of confluence (like in Fig 2E)... In the latter case, mechano-chemical interactions between cells could play a role in calcium signaling. This point should be clarified all along the paper.

 

**Detailed comments**

- Figures 1A, 1B seem to show the culture at higher confluence than 50-70% as used for experiments.
- The linear fits in Fig. 5 A, B, C  don't represent the decay of peaks.

We have read this submission. We believe that we have an appropriate level of expertise to confirm that it is of an acceptable scientific standard.

# Peer review: Referee response for version 1

**DOI:** 10.5256/f1000research.4759.r5433

**Published:** 2014-08-06

**Authors:** Núria Gavara

**Affiliations:** 1 School of Engineering and Materials Science, Queen Mary University of London, London, UK

I've read with interest the manuscript by Lopez-Ayon *at el.* Overall, I think the authors have done a good job in characterizing qualitatively the differences in Ca ^2+^ responses induced by local membrane deformation and micro-injury. I particularly liked the measurements involving cyclic membrane perturbation and inhibition of Ca ^2+^ channels and internal store. I think the latter provide a good understanding on the different origin of the Ca ^2+^ released when cells are probed or micro-injured. Nevertheless, i have a technical concern and some questions that i feel the authors could have discussed more in-depth in the manuscript:

1. Figure 4c and 4e(iv) show large deviations in the deflection of the cantilever when cells are indented for longer time periods. Which kind of feedback was used to control the AFM tip position? It appears as if no deflection feedback was really used, but rather the piezo was kept at constant z. It seems the system is either drifting, or the cell is changing its mechanical properties (softening) or its height (shortening) due to the Ca ^2+^ release. Could the authors comment on that?
2. The fit obtained from fig 4d could be considered to have a slope of 1 (0.98), indicating an on-off response. More interestingly, the offset of 0.6s indicates that no matter the duration of the stimulation (and thus the total Ca ^2+^ released), the time required to recover baseline levels is constant. Would that be an expected behaviour, or is this the case only because the released Ca ^2+^ levels are small and very localized compared to the whole volume cell?
3. In the discussion, the authors suggest that 'intracellular Ca ^2+^ acts as an integrating signal that increases until it reaches the threshold Ca ^2+^ concentration necessary to induce global Ca ^2+^ response'. If that is the case, have they observed any global Ca ^2+ ^responses when using multiple cyclic indentations with very short recovery times? Taking into account their results on Ca ^2+^ released for 1 single indentation, could they predict the number (or duration) of small indentations required to integrate enough Ca ^2+^ signal to start a global Ca ^2+^ response?

I have read this submission. I believe that I have an appropriate level of expertise to confirm that it is of an acceptable scientific standard.
